# Meningococcal carriage and disease—Population biology and evolution

**DOI:** 10.1016/j.vaccine.2009.04.061

**Published:** 2009-06-24

**Authors:** Dominique A. Caugant, Martin C.J. Maiden

**Affiliations:** a WHO Collaborating Centre for Reference and Research on Meningococci, Norwegian Institute of Public Health, Oslo, Norway; b Institute of Oral Biology, Faculty of Dentistry, University of Oslo, Oslo, Norway; c Department of Zoology, University of Oxford, South Parks Road, Oxford OX1 3PS, United Kingdom

**Keywords:** Meningococcal disease, Carriage, Population structure

## Abstract

Meningococcal disease occurs worldwide with incidence rates varying from 1 to 1000 cases per 100,000. The causative organism, *Neisseria meningitidis*, is an obligate commensal of humans, which normally colonizes the mucosa of the upper respiratory tract without causing invasive disease, a phenomenon known as carriage. Studies using molecular methods have demonstrated the extensive genetic diversity of meningocococci isolated from carriers, in contrast to a limited number of genetic types, known as the hyperinvasive lineages, associated with invasive disease. Population and evolutionary models that invoke positive selection can be used to resolve the apparent paradox of virulent lineages persisting during the global spread of a non-clonal and normally commensal bacterium. The application of insights gained from studies of meningococcal population biology and evolution is important in understanding the spread of disease, as well as in vaccine development and implementation, especially with regard to the challenge of producing comprehensive vaccines based on sub-capsular antigens and measuring their effectiveness.

## 1 Introduction

The sole ecological niche of *Neisseria meningitidis* is the mucosa of the oropharynx of humans. Meningococcal colonization of the respiratory tract, a phenomenon commonly referred to as carriage, represents a successful commensal relationship between the host and the bacterium, with the host experiencing no detectable pathology. On the other hand disease represents a failed or dysfunctional relationship with the host [1]. Acquisition of *N. meningitidis* demands person-to-person transmission via direct contact or through dispersion of respiratory droplets from an infected to a susceptible individual. Although often protected by a polysaccharide capsule, meningococci are particularly sensitive to desiccation; thus, spread from one individual to another requires close contact [2]. In closed or semi-closed settings, such as residential schools and military recruit camps, transmission increases dramatically and carriage prevalence may approach 100% [3,4]. While carriage rates are very variable among human populations, point-prevalence carriage rates in Europe and the United States have been estimated to range from 10 to 35% in young adults [5–8] and it is likely that, at one time or another during life, most individuals are colonized with meningococci.

As is the case with other bacterial inhabitants of the mucosa, such as *Haemophilus influenzae* and *Streptococcus pneumoniae*, *N. meningitidis* has a clear pathogenic potential. Shortly after colonization, and usually less than 10 days from first exposure, meningococci can pass through the epithelial cells and enter the blood stream, where they occasionally survive and multiply intravascularly. Progression to severe meningococcal disease can occur very rapidly. The most important factors predisposing individuals to invasive meningococcal disease are the lack of circulating protective bactericidal antibodies and defects in the complement system. From the blood stream the bacterium is then disseminated to various organs and more than half of the patients developing a systemic meningococcal infection will present with clinical symptoms of meningitis [9]. Meningococcal disease is a life-threatening illness and, despite appropriate treatment, the case fatality rate is still around 10% [10].

## 2 Meningococcal carriage

Studies of meningococci isolated from the nasopharynx, which is the normal environment of the meningococcus, are essential to improve knowledge of the epidemiology of meningococcal disease. The results of carriage studies, however, are highly dependent on the swabbing techniques and laboratory methods used. Swabbing of the posterior wall of the oropharynx, followed by immediate cultivation on selective medium, is the recommended procedure to detect asymptomatic meningococcal carriage in an individual [11]. Some real-time PCR methods have been attempted more recently, but their sensitivity is not greater than the microbiological techniques based on culture [12,13].

Age is one of the most important factors influencing meningococcal carriage rates. In Europe and North America, carriage rates are very low in the first years of life, and then sharply increase in teenagers, reaching a maximum in those aged between 20 and 24 years. Carriage rates in older ages are lower than 10% [5,6,8,14]. Studies of meningococcal carriage in Africa, on the other hand, have shown very variable age distributions from one study to another that usually do not correspond to those from Europe and North America [15]. Other factors that increase the risk of being a meningococcal carrier include: male gender; coincident respiratory tract infections of viral or bacterial origin; active as well as passive smoking; and low socio-economic status. One of the most important factors is the number and closeness of social contacts [16].

Most carrier studies are cross-sectional surveys of a target population at a single point in time, i.e. are snapshot studies. In such cases, a positive sample will not identify when the individual acquired the meningococcus and a negative sample might be due to low sampling sensitivity. Some investigators, however, have attempted to measure duration of meningococcal carriage by following subjects with repeated throat samples over time in longitudinal studies. These studies have shown that the meningococcal carrier state may be chronic, lasting for several months, intermittent, or transient [17,18].

To understand the dynamics of transmission of *N. meningitidis* in a population, molecular characterization of the organisms is essential. In most carriage studies, isolate characterization to determine whether the carried strain is the same over time, has either not been performed or the methods employed were relatively insensitive. In the past few years a number of longitudinal studies have employed powerful molecular methods to gain information on the duration of carriage and acquisition rates in populations. These investigations have shown that the commensal association of particular strain with the host is a long-term relationship with 90% of stable carriers keeping the same strain for a period of 5–6 months [19,20]. Longer follow-up periods are required to establish the duration of carriage fully. Duration of carriage depends on the properties of the colonizing strain, and not all meningococcal strains having the same propensity to establish a long-term commensal relationship with a given host.

## 3 Meningococcal disease

Despite high rates of meningococcal carriage in many or most human populations, disease is rare with annual incidence rates that vary from 1 to 1000 cases per 100,000 individuals in different parts of the world. With the exception of patients with complement deficiency, who are predisposed to meningococcal infections, immunocompetent individuals are unlikely to develop the disease more than once.

Meningococcal disease in Europe and North America usually occurs as sporadic cases and the highest age-specific incidence rates are seen in children less than 5 years of age, which is in contrast to the low prevalence of asymptomatic carriage in this age group. The disease may also present different epidemiological features. In some areas hyperendemic disease occurs, with incidence rates of 5–10 cases per 100,000 and these increased incidence waves can last for several decades. Cases can also occur in clusters and localized outbreaks, but the most dramatic epidemiological manifestations are the periodic large countrywide epidemics or pandemics that occur in some parts of the world. Currently, epidemic and pandemic disease appears restricted to countries of sub-Saharan Africa, in the so-called “meningitis belt”, which extends from Ethiopia in the East to Senegal in the West [21].

The capsular polysaccharide is the outermost structure on the meningococcal surface. Of the 12 serogroups identified on the basis of antigenic variation of the capsule, 5 (A, B, C, W135, and Y) are responsible for more than 90% of the invasive disease worldwide and have been associated with hyperendemic and epidemic disease [22]. Recently, a sixth serogroup, serogroup X, has also revealed an epidemic potential [23]. The capsule, which protects the bacterium during the invasion process, is a major virulence factor and the primary target for mucosal and humoral immunity. The currently commercially available meningococcal vaccines target the serogroup A, C, Y and W135 polysaccharides.

The large epidemics in Africa have been, with a few exceptions, associated with serogroup A meningococci, which have essentially disappeared from Europe and North America since World War II [24,25]. Serogroup B meningococci, which are generally absent in sub-Saharan Africa, are the primary concern in industrialized countries, where they have been responsible for hyperendemic waves of disease. Outbreaks of serogroup C meningococcal disease occur worldwide, especially in adolescents and young adults [26] and serogroup Y meningococci have emerged as an important cause of disease in North America in the past 10 years or so [27], while serogroup W135 and X meningococci have been responsible for epidemics in sub-Saharan Africa since 2002 [28].

## 4 Genetic methods for isolate characterization

Multilocus sequence typing (MLST) was first developed in the late 1990s for the meningococcus [29]. It is a high reliable and reproducible characterization method, which assesses variation at multiple genetic loci using nucleotide sequencing. MLST has a very high discriminatory power and the generated data can be readily exchanged among laboratories, through a centralized, curated Internet-accessible database (http://pubmlst.org/neisseria). Developed by a European consortium, the method was readily adopted as the reference genotyping method for *N. meningitidis* by scientists worldwide [30–33]. The success of MLST in application to meningococci resulted in development of similar typing schemes for numerous other bacterial pathogens (see www.mlst.net; www.pubmlst.org).

The general acceptance of MLST over the past of 10 years has enabled the assembly of a public repository of genotypic data representing well over 10,000 meningococcal isolates from both cases of disease and carriers in various parts of the world. This is an extremely valuable source of information relevant to understanding the epidemiology and population biology of the meningococcus. Data from additional genetic methods can be superimposed on the genetic framework provided by MLST to provide further insights into the evolutionary potential of *N. meningitides*—such methods include the sequencing of genes coding for various antigens [34–36] and analyses of patterns of insertion of insertion sequence (IS) elements [37] and variable number tandem repeats (VNTRs) [38,39].

## 5 Genetic structure and evolution of meningococci

### 5.1 Meningococcal genetic variation

The meningococcus is the best-characterized member of the genus *Neisseria* (94% of the sequence types (STs) listed on the PubMLST *Neisseria* database are meningococcal). The species is genetically and antigenically highly diverse: 6751 STs had been assigned at the time of writing. Sequence typing has also identified hundreds of variants and sub-variants of putative vaccine components, such as the PorA outer membrane protein (OMP) (http://neisseria.org/nm/). Extensive genetic exchange among meningococci [40–44] has major implications in combating human disease, both in confusing epidemiological investigations and in complicating vaccine design. Sub-capsular protein variation is especially important, as the development of serogroup B polysaccharide vaccines has been inhibited by poor immunogenicity and fears of inducing autoimmune responses [45].

While meningococcal diversity is extensive, it is highly structured. Studies of variation at housekeeping loci, initially by multilocus enzyme electrophoresis [46] and more recently by MLST [29], had identified 37 groups of closely related meningococci at the time of writing, accounting for 61% of the meningococcal isolates represented in the PubMLST database. These groups, known as clonal complexes, have become the predominant unit of analysis in meningococcal population biology and epidemiology [26]. A minority of clonal complexes, the so-called hyperinvasive lineages, are responsible for a disproportionate number of cases of disease worldwide [26] and can be over-represented in collections of isolates from diseased patients by as much as two orders of magnitude, relative to their prevalence in asymptomatic carriage (Table 1) [47]. One of them, in particular, the ST-11 clonal complex, is remarkable for its very low rates of carriage relative to high incidence of disease [47,48]. Notwithstanding the high levels of horizontal genetic exchange, clonal complexes, especially hyperinvasive lineages, are stable over time with life spans of many decades and during global spread [26]. This stability and the association of clonal complexes with particular antigenic repertoires provides some hope that the development of protein-based vaccines may be possible, if the nature and dynamics of this structuring can be properly understood [35,36,49].

The propensity to cause disease is polygenic, depending on combinations of genes or allelic variants of genes also present in less invasive meningococci [50]. Various attempts have been made to identify the genetic elements that are associated with invasion [51,52], but to date the capsule region, encoding the ability to synthesise a polysaccharide capsule [53], remains the principal ‘virulence determinant’. As a virulence determinant, however, even the role of capsule is ambiguous as only 5 or 6 of the 12 capsule variants are ever associated with a significant number of disease cases. While capsule expression is usually considered necessary (although rare cases caused by non-encapsulated meningococci have been reported in immunocompetent patients [54–56]), it is not sufficient for a meningococcus to cause disease. In contrast to the disease causing strains, approximately 50% of the isolates from healthy carriers do not express a capsule [57]. Further, 16–20% of carried meningococci do not possess the capsular gene region which encodes the genes required to synthesise the capsule [58–60].

In addition to nucleotide sequence variation at shared loci meningococci exhibit extensive variation in gene content. This has been explored by the comparison of whole or partially sequenced genomes [61–65]. Meningococcal genome structure is also diverse in various other ways, including the presence and absence of ISs and large repertoires of repeat elements of various sizes, tracts of repeated nucleotides, and short nucleotide repeats [63,66]. Many of these are involved in mechanisms of gene regulation and at least 65 genes show potential for highly variable gene expression [67]. These ‘contingency genes’ [68] must have evolved as a means of surviving during carriage, perhaps for immune evasion or exploitation of microniches on the mucosal surface. Which genes are expressed and when has a major influence on the development of disease. For example, many meningococci express capsules during transmission, but downregulate this expression during carriage [69]. Invasion of the mucosal epithelium requires the meningococcus to be acapsulate, but once in the bloodstream meningococci must be capsulate to grow to cause bacteraemia. Tropism to the meninges also requires the expression of different genes. The role of differential gene expression during invasion and spread remains to be fully defined.

The existence of defined genetic types with different phenotypes provides the prospect of identifying the genetic traits that are responsible for those phenotypes by genome wide association studies [70] performed with well-defined isolate collections [71]. To date studies of differences in gene content have failed to detect consistent gene content differences among the *Neisseria* species, with the majority of genes shared among the meningococcus, gonococcus and *N. lactamica* [72,73], notwithstanding their consistently different relationships with humans [71]. Genomic studies have, by and large, also failed to identify major differences among meningococci that have not been previously identified by more conventional molecular microbiology [74,75]. Several genome wide association studies have been undertaken within the meningococcus but, as yet, the only new element to be associated with meningococcal disease is a putative phage, identified by whole genome comparisons of disease and carriage isolates [76]. Intriguingly, although this element is associated with particular clonal complexes, it has a measurable effect on the likelihood of a meningococcus causing disease, independent of this association. Further this effect is predominantly seen in meningococci isolated from teenagers—the element is underrepresented in isolates from younger children and over-represented in adolescents [77]. With the advent of novel high through-put parallel sequencing, this ‘population genomics’ approach is likely to provide further insights into meningococcal biology in the immediate future [71].

### 5.2 Meningococcal evolution

The meningococcus, in common with other members of the genus *Neisseria*, is naturally competent for transformation by exogenous DNA and studies of horizontal genetic exchange among these bacteria [78–82] played an important role in the development of models of bacterial speciation and population structure at the sub-species level. These studies are also important in understanding meningococcal epidemiology and virulence. High observed recombination rates in meningococcal populations, together with the fact that as the *N. meningitidis* is primarily a commensal organism that gains no advantage from invasion of the host, poses two paradoxes: (i) how can clonal complex structure emerge and persist without being broken down by frequent recombination? and (ii) how can some of these clonal complexes be associated with a propensity to cause disease, as meningococci that do not harm their hosts should be favoured during asymptomatic transmission? These paradoxes have to be addressed by models of meningococcal evolution and, given that invasion of the host is an evolutionary dead-end, such models need to be tested with data obtained from carried populations of meningococci.

Although recombination has attracted interest as a mechanism for generating diversity [83], much – indeed most – genetic exchange is almost certainly among very closely related meningococci (i.e., sister cells), and will therefore be conservative, homogenising, and normally unobservable, as it will involve the replacement of a segment of the genome with an identical segment. Support for this view has come from the observation of a strong link between recombination and *Neisseria* DNA uptake sequences (DUS). These short sequences, which promote the uptake of *Neisseria* DNA by other *Neisseria*, are concentrated in regions of the genome that encode DNA maintenance and other ‘core’ *Neisseria* genes [84] suggesting that they may be important in genome stability [85]; if their role was principally in generating diversity it would be expected that they would be sparse in core conserved genes and common in highly variable genes.

The distribution of DUS is therefore consistent with recombination being primarily a mechanism for genome repair that can occasionally result in generation of diversity, which, even more occasionally, is adaptive. It may be that this repair function is especially important in the *Neisseria*, which lack several DNA repair genes [66]; this is also consistent with the distribution of restriction modification systems among meningococci, with particular systems associated with given clonal complexes [86]. The restriction modification systems may therefore act to promote genetic exchange among very close relatives while reducing (but not absolutely preventing) genetic exchange among meningococci belonging to different clonal complexes and related species.

Clonal complex structure, which is such a feature of meningococcal populations, can be explained by models of clonal descent with periodic selection [87], but such models are not consistent with the lack of a clonal phylogeny of meningococci [42], and high observed rates of recombination [44]. The ‘epidemic’ [88] and, later, ‘neutral microepidemic’ models [89] of population structure were developed to accommodate clonal complex structure in recombining populations. These models envisage structure in meningococcal populations reflecting short-term dominance of particular clones and can explain the patterns of variation seen in cross-sectional surveys quite well [89]. These models cannot, however, explain the persistence of clonal complex structures or the association of some lineages with the hyperinvasive phenotype [90].

Alternative dynamic models explore the organisation of populations into strains in the context of selective forces [91]. Structuring of antigen variants encoded at multiple loci can be explained in meningococci, and other recombining pathogens such as *Plasmodium falciparum*, by immune selection acting on them. Depending on the intensity of positive selection, i.e., the strength of the immune interactions with the antigens, a range of different population structures can be generated. Importantly, the antigenic repertoires of strains generated by this mechanism will be characteristically non-overlapping, in that the different strains circulating in the population will not share variants at each of the loci under selection. Such non-overlapping repertoires have been observed in the meningococcal surface genes, especially the porins and Opa proteins [90,92,93]. The consequent limitations of the repertoires of such antigens available to circulating meningococci therefore have major implications for rational vaccine design [35,36]. These immunological models can also explain clonal complex structure if the combinations of housekeeping genes were hitch-hiking with genes encoding the antigenic variants repertoire; however, the antigenic structuring although mirroring clonal complex, is not always congruent with it, with some members of the same clonal complex exhibiting different antigenic repertoires and occasional examples of the same antigen occurring in different clonal complexes. Combined with high rates of recombination, this makes hitch-hiking an unlikely explanation for clonal complex structure.

A major limitation of all of the models discussed so far is that they provide no explanation for the association of certain clonal complexes with an increased propensity to cause disease. If, however, the clonal complexes are regarded as units of selection, with particular STs being associated with fitness for transmission, both clonal complex structure and the hyperinvasive phenotype can be explained within the context of competition for hosts among different meningococcal genotypes [90]. This insight has the important implication that the observed genetic structuring must have a phenotypic consequence. If this is the case, STs are subject to selection and not neutral markers as previously thought; the model further demonstrates that fitness differences among distinct types are very small [90]. These insights are complementary to a stochastic model of meningococcal disease outbreaks, which showed that for large disease outbreaks, very small differences in pathogenic potential are necessary [94]. Diversity, and the forces which structure it, therefore appear to be central to the biology and pathogenicity of meningococci.

## 6 Implications for vaccine design

Meningococcal disease remains incompletely controlled by immunisation, largely as a consequence of the diversity of *N. meningitidis* populations. In terms of vaccine candidates, the existence of five, or at most six different polysaccharide capsules is not a challenge, given the successful implementation of conjugate polysaccharide vaccines available against pneumococci that contain multiple components [95]. However, the fact that the meningococcal serogroup B capsular antigen is chemically similar to the host antigen NCAM [96] has raised safety concerns that have precluded the development of comprehensive capsular vaccines against the meningococcus [45].

The immunology of conjugate polysaccharide vaccines that target meningococcal capsules was thought to be well understood at the time of their introduction into national immunisation programs [97,98]. The conjugation of the polysaccharide to a protein elicits a T-cell-dependent immune response and immunological memory, both of which are absent when pure polysaccharide is used as an immunogen. Studies of carriage during vaccine introduction in the United Kingdom, together with continued disease surveillance in countries where the vaccines have been introduced, have demonstrated an additional powerful herd immunity effect. As well as providing strong personal protection, meningococcal conjugate vaccines protect the population, including unvaccinated individuals, by interrupting the transmission of capsulate meningococci [99,100]. Intriguingly in the UK this effect was strongly directed at serogroup C meningococci belonging to the ST-11 complex and made a major contribution to the success of this vaccine introduction [48]. The magnitude of this effect could be attributed to the inclusion of teenagers, among whom most meningococcal transmission occurs, in the immunisation campaign [16]. Carriage studies therefore provide an additional way of monitoring the effectiveness of immunisation programmes, as well as indicating the most efficient implementation strategies.

Although providing an apparent alternative to conjugate polysaccharides, especially in the case of serogroup B meningococci, the sub-capsular antigens provide challenges of their own [45]. Where sub-capsular vaccines, particularly the outer membrane vesicle vaccines, have been successful this has been a consequence of their effectiveness against particular hyperinvasive lineages, and their design has been based on epidemiological knowledge of the prevalent disease causing lineages [101–103]. These approaches will, however, have a limited impact on disease caused by a variety of hyperinvasive meningococci, such as its typical of endemic disease in Europe and North America [104,105], and there is little evidence of effective herd immunity induced by such vaccines. Therefore, if sub-capsular vaccines are to be effective against a broad range of meningococci, they will either have to be based on major surface components that are antigenically invariant and reliably expressed in all or most disease-associated meningococci (if such components indeed exist, which remains an open question) or they will have to contain cocktails of vaccine antigens carefully formulated on the basis of the molecular epidemiology of the meningococcus [106]. In either case, knowledge of the molecular epidemiology and evolution of disease and carried meningococci will be central to the design implementation and assessment of such vaccines.

In conclusion, studies of the carrier state of the meningococcus remain of central importance in combating this important pathogen. Understanding the spread of invasive meningococci depends on appreciating the natural history of the organism and defining the dynamics of asymptomatic transmission. Resolving the apparent paradox of meningococcal virulence depends on refining models of meningococcal evolution, a process that occurs exclusively during carriage and transmission. Finally, the design and optimal use of meningococcal vaccines depends on acknowledging their effect on carriage. While much had been learned concerning each of these subjects over the last decade, much remains to be elucidated in this intriguing and important area of meningococcal biology.

## Figures and Tables

**Table 1:** Characteristics of the most important clonal complexes of *Neisseria meningitidis* (data compiled from the PubMLST database 6/02/09).

ST-complex	MLEE designation	No. isolates	No. STs	Dominant serogroups (%)	Dominant PorA	Dominant FetA	Disease/carriage ratio^a^	Main origin
ST-1 complex	Subgroup I/II	204	49	A(97)	5-2,10	F3-5	5.5	Russia, China
ST-5 complex	Subgroup III	627	33	A(99)	20,9	F3-1	19.5	Africa
ST-8 complex	Cluster A4	283	107	B(51),C(35)	5-1,2-2	F3-6	24.5	Europe
ST-11 complex	ET-37 complex	1142	239	C(57),W135(24),B(12)	5,2	F3-6	6.6	Worldwide
ST-18 complex	Cluster J1	208	175	B(85)	22,14	F3-6	5.5	Czech Republic, Poland
ST-22 complex		363	243	W135(52),NG(25)	18-1,3	F4-1	0.6	UK
ST-23 complex	Cluster A3	385	154	Y(62),NG(18)	5-1,2-2	F4-1	0.8	Worldwide
ST-32 complex	ET-5 complex	1028	350	B(85)	19,15	F5-1	3.5	Worldwide
ST-35 complex		329	214	B(59),NG(25)	22-1,14	F4-1	0.5	Worldwide
ST-41/44 complex	Lineage 3	1796	1274	B(70)	7-2,4	F1-5	1.2	Worldwide
ST-53 complex		272	93	NG(76)	7-2,30	F1-7	<0.1	UK
ST-60 complex		225	148	B(30),29E(22),NG(19)	5,2	F1-7	0.7	Europe
ST-103 complex		127	84	B(26),NG(22),C(16)	18-1,3	F3-9	1.2	Worldwide (-Africa)
ST-162 complex		140	63	B(74),NG(13)	22,14	F5-9	0.8	Worldwide
ST-167 complex		201	144	Y(47),NG(36)	5-1,10-4	F3-4	0.5	Worldwide
ST-198 complex		166	76	NG(76)	18,25-15	F5-5	<0.1	Worldwide
ST-213 complex		187	165	B(74),NG(16)	22,14	F5-5	0.6	UK
ST-254 complex		148	107	NG(35),B(24),29E(12)	5-1,16	F1-7,F3-6	0.5	Worldwide
ST-269 complex		415	312	B(73)	22,9	F5-1	2.8	Worldwide
ST-334 complex		106	64	C(58),B(33)	5-1,2-2	F1-5	5.7	UK

a This ratio is calculated on from data deposited the PubMLST database, which contains only those isolates submitted to it by members of the *Neisseria* research community. Although it represents a comprehensive overview of the diversity observed to date, this database is not a coherent population sample. More precise calculations of the ratio of disease to carrier ratios for particular populations are available in Refs. [47,77].
